# Surveillance of life-long antibiotics: a review of antibiotic prescribing practices in an Australian Healthcare Network

**DOI:** 10.1186/s12941-017-0180-6

**Published:** 2017-01-18

**Authors:** Jillian S. Y. Lau, Christopher Kiss, Erika Roberts, Kylie Horne, Tony M. Korman, Ian Woolley

**Affiliations:** 1 Monash Infectious Diseases, Monash University, Monash Health, Clayton, VIC Australia; 2 Monash Health Pharmacy, Monash Health, Clayton, VIC Australia

**Keywords:** Antibiotics, Resistance, Infection, Suppression, Pharmacy

## Abstract

**Background:**

The rise of antimicrobial use in the twentieth century has significantly reduced morbidity due to infection, however it has also brought with it the rise of increasing resistance. Some patients are on prolonged, if not “life-long” course of antibiotics. The reasons for this are varied, and include non-infectious indications. We aimed to study the characteristics of this potential source of antibiotic resistance, by exploring the antibiotic dispensing practices and describing the population of patients on long-term antibiotic therapy.

**Methods:**

A retrospective cross-sectional study of antibiotic dispensing records was performed at a large university hospital-based healthcare network in Melbourne, Australia. Outpatient prescriptions were extracted from the hospital pharmacy database over a 6 month period in 2014. Medical records of these patients were reviewed to determine the indication for prescription, including microbiology, the intended duration, and the prescribing unit. A descriptive analysis was performed on this data.

**Results:**

66,127 dispensing episodes were reviewed. 202 patients were found to have been prescribed 1 or more antibiotics with an intended duration of 1 year or longer. 69/202 (34%) of these patients were prescribed prolonged antibiotics for primary prophylaxis in the setting of immunosuppression. 43/202 (21%) patients were prescribed long-term suppressive antibiotics for infections of thought incurable (e.g. vascular graft infections), and 34/43 (79%) were prescribed by Infectious Diseases doctors. 66/202 (33%) patients with cystic fibrosis were prescribed prolonged courses of macrolides or fluoroquinolones, by respiratory physicians. There was great heterogeneity noted in indications for prolonged antibiotic courses, as well as antibiotic agents utilised.

**Conclusion:**

Our study found that that continuous antibiotic therapy represented only a small proportion of overall antibiotic prescribing at our health network. Prolonged courses of antibiotics were used mainly to suppress infections thought incurable, but also as primary and secondary prophylaxis and as anti-inflammatory agents. More research is needed to understand the impact of long-term antibiotic consumption on both patients and microbial ecology.

## Background

Antibiotics have revolutionised the treatment of acute infection, including making many previously life-threatening diseases potentially survivable. With the discovery of penicillin [1], and the rapid development of antibiotics in the second half of the twentieth century, morbidity and mortality associated with bacterial diseases has significantly fallen [2]. Antibiotic agents are usually prescribed for acute infection. Although there are very few studies of efficacy, long term antibiotics are used for three distinct purposes; prophylaxis (primary or secondary), long term suppression of existing infections and for non-antibiotic effects like immunomodulation, or prokinetic effects [3–12].

With the rise of the use of antibiotics in humans and agriculture, has come the increasing scourge of antibiotic resistance [13, 14]. In principle, long-term antimicrobial use might play a role in the development of resistance. This population of patients has not been well studied, and it is not known what proportion of overall antimicrobial prescribing is intended to be for long-term courses. We wished to examine the number of patients on long-term antibiotics, review their indications, and to see if they might be so large a proportion as to play a role in the development of antibiotic resistance.

The aim of this analysis was to describe the use of long-term antibiotics in hospital outpatients, and to determine how many patients were on long-term suppressive antibiotic therapy. An analysis of these patients including indication (medical condition, microbiology) and antimicrobial agent and dose, was performed, to describe in detail this previously unstudied, yet important population of patients.

## Methods

A retrospective, cross-sectional study was performed of the dispensing episodes of antimicrobial agents at Monash Health, an integrated health network in Melbourne, Victoria, Australia, which comprises 6 public hospitals with 2130 acute, sub-acute, mental health and aged care beds, 19 community health service centres and residential care facilities, In 2013–14, 843,162 patients received care as outpatients at specialty clinics including endocrinology, neurosurgery, rheumatology, renal transplant and orthopaedics [15].

Eligible patients were identified using the hospital drug management system (Merlin Ver. 4.94, Pharmhos Software, Port Melbourne, Victoria, Australia). All outpatient prescriptions of antimicrobial agents over a 6 month period (February–July 2014) were extracted and this data was entered in Microsoft Excel (Microsoft Corp, Seattle WA, 2015). The dispensing records were filtered for those with at least one repeat, oral formulations (including tablets, capsules and granules), and antibacterial agents (using the WHO Collaborating Centre for Drug Statistics ATC classification system). Inpatient prescriptions, intravenous orders, topical or non-oral formulations were excluded. Patients were identified who were prescribed antibacterial agents with an intended duration of 1 year or greater. Medical records were reviewed to determine patient demographics, the indications for prescription including microbiology, the intended duration, and the prescribing doctor specialty. A descriptive analysis was performed on this data.

Ethics approval was granted for the project by the Monash Health Human Research Ethics Committee, and individual patient consent was not required for this retrospective study.

## Results

Of a total of 66,127 outpatient prescriptions, 514 patients were prescribed one or more oral antibacterial agents, and 202 outpatients fulfilled our study criteria, identified treated with an intended duration of 1 year or greater.

Three distinct indications for prolonged antibiotic therapy were identified: primary prophylaxis in the context of immunosuppression, secondary prophylaxis for infections thought incurable, and long-term antibiotics for “other” reasons (see below).

Sixty-nine (34%) of the 202 patients were prescribed prolonged antibiotic courses for primary prophylaxis for *Pneumocystis jirovecii* pneumonia (PJP), and post splenectomy. PJP prophylaxis with trimethoprim/sulfamethoxazole was prescribed in the context of immunosuppression by various specialties including Renal Transplant (n = 1), Haematology (n = 12), Oncology (n = 41), Rheumatology (n = 1), and Infectious Diseases (n = 9). Five patients were prescribed antibiotics for splenectomy prophylaxis: amoxicillin (2), erythromycin (1), phenoxymethylpenicillin (1), and roxithromycin (1).

**Fig. 1:**
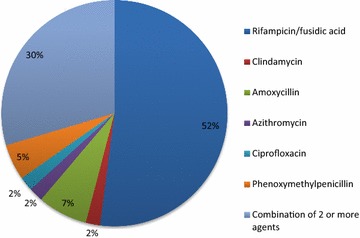
The most commonly prescribed antibiotics for the purpose of suppressing incurable infections

Of the 202 patients prescribed prolonged antibiotic courses, 43 (21%) were for long-term suppressive therapy, with over half (n = 25) for infected prosthetic devices (e.g. joint replacements, spinal fixation devices), and also chronic bone infections without prosthetic material (n = 6), vascular graft infections (n = 6) and infected cardiac devices (n = 6). Antibiotic agents commonly prescribed were rifampicin, in combination with fusidic acid, amoxicillin, and phenoxymethylpenicillin. Combination therapy with 2 or more agents were also utilised (Fig. 1).

These antibiotics were predominantly prescribed by Infectious Diseases doctors (n = 34), but also by clinicians from Cardiology (n = 1), Rheumatology (n = 1), General Medicine (n = 4) and Orthopaedics (n = 1). In all the cases where patients were prescribed antibiotics by other units, Infectious Diseases doctors had been consulted for at least the initial prescription. The most common organism isolated was methicillin-resistant *Staphylococcus aureus* (MRSA) (n = 22, 54%). Other organisms included methicillin susceptible *S. aureus* (MSSA) (n = 4), coagulase-negative staphylococci, streptococci and *Propionibacterium acnes*. Eight patients were prescribed treatment for infections where multiple organisms had been isolated.

The “other” group consisted of patients prescribed prolonged antibiotics for difficult to treat infections (with a curative intent), such as mycobacterial infections. Patients prescribed antimicrobial agents for anti-inflammatory purposes, and patients prescribed secondary prophylaxis for urinary tract infections (n = 5).

66/202 (33%) patients with cystic fibrosis were prescribed prolonged courses of macrolides or fluoroquinolones by respiratory physicians. A planned duration was not documented for 63 of 66 (95%) of patients. 20/202 (10%) patients with cystic fibrosis were prescribed prolonged antibiotic therapy for complex mycobacterial infection, including *Mycobacterium tuberculosis* (n = 8), *Mycobacterium avium complex* (n = 8), *Mycobacterium leprae* (n = 2), *Mycobacterium abscessus* (n = 1) and *Mycobacterium haemophilum* (n = 1).

Table 1 describes the patient demographics in each of the patient groups where antibiotics are used for primary or secondary prophylaxis. Compared to the other indications for prolonged antibiotics, patients with prosthetic device-associated infections (including orthopaedic prostheses, cardiac devices and vascular grafts) were older than patients on prolonged prophylaxis. Infectious Diseases consultation was also sought more often in these groups.

**Table 1:** Patient Demographics by indication for prolonged antibiotic therapy

Indication (number)	Age	Gender	Infectious diseases
	Median (SD)	Male (%)	Consultation (%)
Splenectomy (5)	26 (21)	3 (60)	1 (20)
PJP prophylaxis (69)	16 (25.1)	35 (55)	9 (14)
Prosthetic device infection (25)	77 (9.6)	13 (52)	23 (92)
Vascular graft infection (6)	81.5 (7.5)	3 (50)	5 (83)
Osteomyelitis/septic arthritis (6)	65.5 (3.7)	4 (67)	4 (67)
Implantable cardiac device (6)	65 (19.4)	3 (60)	3 (50)

PJP, *Pneumocystis jirovecii* pneumonia; SD, standard deviation

## Discussion

We reviewed 66,127 prescriptions dispensed from our health network over a 6 month period, and identified 202 patients prescribed long-term antibiotics, defined as continuous usage of over 1 year. Prolonged courses were prescribed in the setting of primary prophylaxis, secondary prophylaxis in infections thought incurable, and in complex or difficult to treat infections. There were also a number of cystic fibrosis patients who were prescribed long-term macrolides or fluoroquinolones for anti-inflammatory rather than anti-infective purposes.

This is the first study to review the long-term antibiotic prescribing practices in a tertiary healthcare network. With this data we have been able to identify the clinicians who prescribed long-term antibiotics courses, and for which indications. Infectious diseases doctors did not prescribe the majority of courses of prolonged antibiotics seen in our review, and a planned duration was often not documented.

We have also described the population of patients consuming these antibiotics, including their demographics, and the microorganisms that the antibiotics were targeting.

However, our retrospective study, using administrative data sets, may have underestimated use of antibiotics in some populations. For example, we only identified one patient under prescribed trimethoprim-sulphamethoxazole prophylaxis by our renal transplant unit. We had anticipated a higher number of prescriptions for PJP prophylaxis post solid organ transplant as per local and international guidelines [4, 16, 17]. On review of the renal unit prescribing practices, it was noted that prescriptions of only 10 tablets with 1 repeat were dispensed to ensure frequent follow up, and hence these patients were missed by our study methods.

Only four patients were prescribed flucloxacillin or cephalexin for long-term suppression of infections with methicillin sensitive *S. aureus*, a common cause of orthopaedic and other infections treated at our healthcare network [18]. These antibacterial agents are more likely to be prescribed by primary care clinicians and/or dispensed by community pharmacies via the Australian Pharmaceutical Benefits Scheme (PBS). The common prescription of rifampicin for treatment of chronic MRSA infection can be explained by the lack of availability for subsidised prescribing of this expensive agent in the community for this indication.

No patients were prescribed prolonged or prophylactic courses of antibiotics for recurrent chest infections which is not recommended in evidence based guidelines [8, 19, 20]. Clinicians may be prescribing these antibiotics outside of the public hospital outpatient setting (i.e. private consulting rooms) and/or dispensed in community pharmacies.

The proportion of patients on long-term antibiotics relative to the total number of dispensed prescriptions was low, reflecting a very small number of patients taking antibiotics for a year or longer. We anticipate that this population may not be important in overall antibiotic usage and the development of antimicrobial resistance. However while our review has defined this group of patients, little has been studied about the long-term effects of taking prolonged courses of antibiotics, from side effect burden, to alteration of the microbiome on an individual and population level. Further studies are needed to explore these gaps in knowledge.

## Conclusion

The heterogeneity in indications and antibiotics prescribed highlights the lack clear guidelines based on well-designed randomised controlled trials, particularly relating to suppression of infections thought incurable. More research is needed to understand the impact, on an individual and population level, of long-term antibiotic consumption, in order to design appropriate guidelines for the prescribing and monitoring of life-long antibiotic therapy.

## Abbreviations

ATC: Anatomical Therapeutic Chemical Classification System
ID: infectious diseases
MSSA: methicillin sensistive *Staphylococcus aureus*
MRSA: methicillin resistant *Staphylococcus aureus*
PJP: *Pneumocystis jirovecii* pneumonia
